# COVID-19-Triggered Acute Exacerbation of IPF, an Underdiagnosed Clinical Entity With Two-Peaked Respiratory Failure: A Case Report and Literature Review

**DOI:** 10.3389/fmed.2022.815924

**Published:** 2022-02-03

**Authors:** Yosuke Goto, Koji Sakamoto, Jun Fukihara, Atsushi Suzuki, Norihito Omote, Akira Ando, Yuichiro Shindo, Naozumi Hashimoto

**Affiliations:** Department of Respiratory Medicine, Nagoya University Graduate School of Medicine, Nagoya, Japan

**Keywords:** COVID-19, acute exacerbation, idiopathic pulmonary fibrosis, high resolution CT scan, viral infection

## Abstract

Because severe coronavirus disease 2019 (COVID-19) affects the respiratory system and develops into respiratory failure, patients with pre-existing chronic lung disorders, such as idiopathic pulmonary fibrosis (IPF), are thought to be at high risk of death. Patients with IPF often suffer from a lethal complication, acute exacerbation (AE), a significant part of which is assumed to be triggered by respiratory viral infection. However, whether mild to moderate COVID-19 can trigger AE in patients with IPF remains unknown. This is the case report of a 60-year-old man with a 4-year history of IPF who successfully recovered from moderate COVID-19 but subsequently developed more severe respiratory failure, which was considered to be a COVID-19-triggered acute exacerbation of idiopathic pulmonary fibrosis (AE-IPF). It is important to be aware of the risk of AE-IPF after COVID-19 and to properly manage this deadly complication of IPF. Recent literature reporting cases with chronic interstitial lung diseases which developed respiratory failure by complications with COVID-19 is also reviewed and discussed.

## Introduction

Since its emergence in December 2019, more than five million patients have died of coronavirus disease 2019 (COVID-19) (1), which is caused by infection with severe acute respiratory syndrome coronavirus 2 (SARS-CoV-2) and manifests as acute respiratory failure in severe cases. Pre-existing chronic lung diseases have a significant impact on the clinical course and outcomes of patients with COVID-19 (2). Among those lung diseases, a significant increase in mortality due to COVID-19 has been reported in patients with idiopathic pulmonary fibrosis (IPF) (3). In particular, acute exacerbation (AE), a devastating complication of IPF (4), may be triggered by COVID-19 and can be one of the major causes of this elevated mortality. However, because severe COVID-19 and AE-IPF share many clinico-radiological features, it is not easy to distinguish these two diseases definitively, and there have been few reports clearly demonstrating the clinical course of AE-IPF induced by COVID-19.

We here present the clinical course of a case with pre-existing IPF presenting with COVID-19 that was successfully treated and recovered, but subsequently developed AE-IPF with severe and prolonged respiratory failure that was considered to be triggered by COVID-19. We believe it is important to understand the clinical course of this newly recognized trigger of AE-IPF in the era of the COVID-19 pandemic.

## Case Presentation

**Figure 1:**
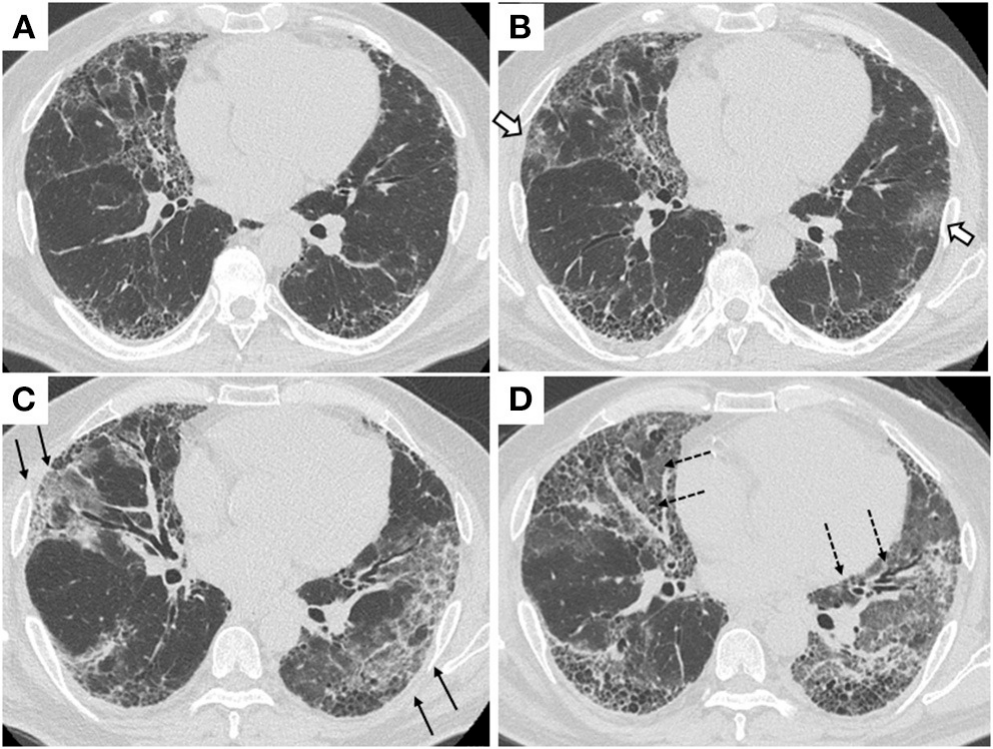
Chest CT scans. **(A)** On his last regular visit prior to the first admission. **(B)** On his first admission due to COVID-19. Open arrows indicate newly developed ground-glass opacities in the outer zones of both lungs. **(C)** On day 15 of his first admission, just before discharge. Arrows indicate ground-glass opacities were increased but limited to the outer zones of both lungs. **(D)** On his re-admission due to AE-IPF. Broken arrows indicate extensive ground-glass opacities spread to the inner zones of both lungs.

A 60-year-old Japanese man with a medical history of pulmonary tuberculosis, mild cerebral infarction, and type 2 diabetes mellitus presented at our hospital after 4 days of fever and pharyngeal discomfort. He had been diagnosed with IPF and treated with nintedanib for 4 years. He was not vaccinated against SARS-CoV-2. On presentation, he did not complain worsening dyspnea, cough, or sputum. On examination, his body temperature was 36.0°C, respiratory rate was 24/min, and oxygen saturation was 97% in room air, and he had not deteriorated since his last periodic visit. Compared to his baseline chest computed tomography (CT) (Figure 1A), that on admission demonstrated newly developed ground-glass opacities (GGOs) in the right middle and left upper lobes overlaying the pre-existing bilateral reticular abnormalities and honeycombing predominantly distributed in the subpleural area (Figure 1B). The real-time polymerase chain reaction (RT-PCR) test for SARS-CoV-2 performed on admission was positive. Blood test results are shown in Table 1. Based on a diagnosis of mild COVID-19, he was admitted to the hospital according to the governmental policy at that time and treatments for possible concomitant bacterial infection (ceftriaxone and azithromycin) were initiated. On day 6 of admission, dexamethasone and favipiravir were initiated because he had developed respiratory failure requiring 1 L/min of oxygen via a nasal cannula. His respiratory status gradually improved after the initiation of anti-COVID-19 treatment and supplemental oxygen was successfully withdrawn on day 16 of admission. After two negative salivary SARS-CoV-2 RT-PCR tests on consecutive days, he was ambulatory and discharged 22 days after admission. A chest CT scan taken just before discharge revealed that peripheral GGOs were mildly extended compared to the scan at admission (Figure 1C). Because his respiratory status was significantly improved, we considered this was due to delayed absorption of infiltrates.

**Table 1:** Blood test findings of the case.

**Variable**	**Reference range**	**COVID-19 on admission**	**AE-IPF on admission**
White-cell count (/μL)	3,300–8,600	5,400	8,800
Differential count			
Neutrophils (%)	36–74	56.0	63.0
Lymphocytes (%)	14–55	30.3	26.6
Hemoglobin (g/dL)	13.7–16.8	13.6	13.4
Hematocrit (%)	40.7–50.1	41.3	40.7
Platelet count (/μL)	158,000–348,000	214,000	201,000
Sodium (mmol/L)	138–145	139	140
Potassium (mmol/L)	3.6–4.8	3.3	3.0
Chloride (mmol/L)	101–108	105	105
Urea nitrogen (mg/dL)	8.0–20.0	10.4	10.6
Creatinine (mg/dL)	0.65–1.07	0.63	0.91
Glucose (mg/dL)	73–109	219	317
Alanine aminotransferase (U/L)	10–42	13	21
Aspartate aminotransferase (U/L)	13–30	24	29
Alkaline phosphatase (U/L)	106–322	154	214
Total protein (g/dL)	6.6–8.1	5.9	5.5
Albumin (g/dL)	4.1–5.1	2.9	2.3
Creatine kinase (U/L)	59–248	253	154
Lactate dehydrogenase (U/L)	124–222	280	512
Procalcitonin (ng/mL)	<0.5	–	<0.1
C-reactive protein (mg/dL)	≤ 0.14	2.95	17.58
Ferritin (ng/mL)	22–275	1,375	835
D-dimer (μg/mL)	<1.0	0.54	2.20
Krebs von den Lungen-6 (U/mL)	<500	691	1,545

**Figure 2:**
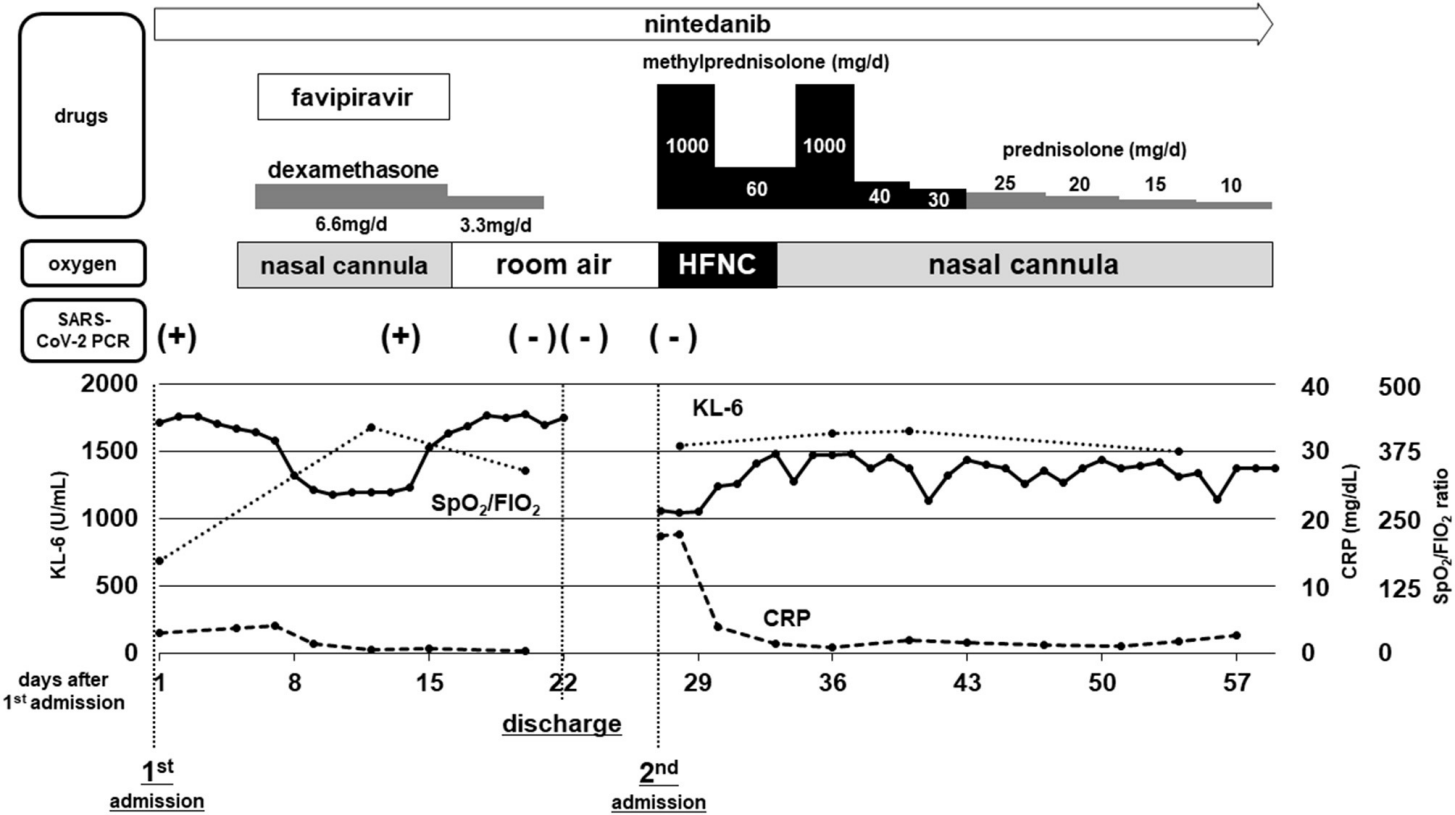
Schematic chart of clinical course and treatment. Therapeutic regimen and the dosages of drugs for COVID-19 and IPF/AE-IPF are shown at the top. The methods of oxygen supplementation required for the patient and the results of SARS-CoV-2 RT-PCR tests are shown in the middle. Changes in serum levels of C-reactive protein (CRP) and Krebs von den Lungen-6 (KL-6), along with the oxygenation index (ratio of SpO_2_/FIO_2_) are depicted in the bottom graph.

After discharge, the patient had a gradual worsening of dyspnea on exertion and re-visited our hospital 5 days later. On arrival at the emergency department, the patient was in respiratory distress with a respiratory rate of 30/min and an oxygen saturation of 95% on supplemental oxygen at a rate of 4 L/min using a face mask. Chest CT showed that bilateral diffuse ground-glass opacities extended to the inner zones (Figure 1D). The blood test showed remarkable elevations of lactate dehydrogenase (LDH) (512 U/L), C-reactive protein (CRP) (17.58mg/dL), and Krebs von den Lungen-6 (KL-6, a biomarker for fibrotic interstitial lung diseases (ILDs) (1,545 U/mL; Table 1). A salivary SARS-CoV-2 RT-PCR test on re-admission was negative. No significant bacterial pathogens were isolated from sputum cultures, and no physical signs suggestive of heart failure were observed. A diagnosis of AE-IPF was made based on the clinical and radiological findings. He was admitted to the ICU and given high-flow nasal cannula oxygen therapy. Steroid pulse therapy (methylprednisolone 500 mg twice daily, 3 consecutive days/week/cycle), tazobactam/piperacillin, and azithromycin were started (Figure 2). After 6 days of respiratory failure which required high-flow nasal cannula therapy, his respiratory status slowly improved with 2 cycles of steroid pulse therapy and subsequent methylprednisolone was gradually reduced and replaced with oral prednisolone. However, persistent hypoxia requiring supplementary oxygen continued on his discharge on the 33rd day of the second admission.

## Discussion

Like patients with several other pre-existing medical conditions (5, 6), those with underlying lung comorbidities including chronic obstructive pulmonary disease, asthma, and ILDs have been shown to be at increased risk of severe disease and mortality due to COVID-19 (2). Previous studies have revealed that COVID-19 patients with pre-existing ILDs, especially fibrotic ILDs, are at very high risk of death (3, 7). Drake et al. assessed the relationship between pre-existing ILDs and mortality in patients with COVID-19 and showed that decreased lung function and a diagnosis of IPF (rather than other ILDs), both of which have also been reported as risk factors of developing acute exacerbation of pre-existing ILDs, were risk factors of death among ILD patients (3). Therefore, one of the reasons for a poor prognosis of COVID-19 in ILD patients is assumed to be acute exacerbation of interstitial lung disease (AE-ILD). In fact, a Japanese nationwide survey reported 12 suspected cases of AE-ILD associated with COVID-19 (8). However, this lethal complication is difficult to distinguish from severe COVID-19 on the basis of clinical manifestations.

The definition of AE-IPF proposed by the international working group is widely accepted currently (4). According to this, AE can be subcategorized into “triggered AE” which is followed by a known predisposing factor such as infection and invasive procedures, and “idiopathic AE” with no trigger identified. It has been suggested that respiratory viral infection triggers AE-ILD (9). While the exact mechanisms of AE development remain unknown, a systemic inflammatory insult with elevated cytokine levels is thought to be a central part of its pathogenesis. Similarly, SARS-CoV-2 infection also induces excessive host immune response, the so-called “cytokine storm” in severe cases. Therefore, although not yet proven statistically, it is suspected that COVID-19 may be one of the common triggers of AE in ILD patients in the current pandemic.

Typical severe COVID-19 exhibits sudden respiratory failure with newly developed bilateral lung opacities on chest CT, which are also common in AE-ILDs. Indeed, according to recent reports demonstrating cases of acute respiratory failure in patients with pre-existing chronic ILDs (10–12), it is hard, if not impossible, to distinguish severe COVID-19 from AE-ILDs triggered by COVID-19. In this case, we observed extension of peripheral dominant GGOs at the time of clinical recovery from COVID-19 (Figure 1C). We deemed them radiological findings of delayed recovery. A retrospective study suggested lung abnormalities on chest CT scans showed the greatest severity around 10 days and gradual resolution 2 weeks after the initial onset of symptoms (13). However, it is also possible that the extension of GGOs shown on chest CT taken at his initial discharge could be the radiological findings of undiagnosed AE-IPF. Indeed, peripheral dominant GGOs are one of the typical subtypes for the radiological feature of AE-IPF (14, 15).

As the pandemic of COVID-19 being prolonged, increasing number of cases with chronic ILDs who developed severe respiratory failure by complication with COVID-19 has been reported. We reviewed and summarized them in Table 2 (8, 11, 12, 16–19). The majority of the cases developed respiratory failure with newly-emerged GGOs simultaneously with the diagnosis of COVID-19, therefore treated with combination of antiviral and immunomodulation therapy against COVID-19. In these cases, it is hard to differentiate between “severe COVID-19 superimposed on pre-existing ILDs” and “AE of chronic ILDs triggered by SARS-CoV-2 infection.” The present case showed the characteristic “two-peak” course (Figure 2) of respiratory failure in the initial COVID-19 phase, with successful recovery due to dexamethasone and favipiravir, and a negative RT-PCR test. The second peak with a more profound and persistent illness can be recognized as infection-triggered AE-IPF. We believe this case is noteworthy as it is important to understand, but not well-reported, the natural course of development of AE clearly triggered by COVID-19. A few reports also described the delayed onset of profound worsening of chronic ILDs after mild COVID-19 (20, 21).

**Table 2:** Summary of reported cases with chronic ILDs who developed respiratory deterioration by complications with COVID-19.

**References**	**Age, yr**	**Sex**	**Smoking**	**Diagnosis of ILD**	**Antifibrotic drugs**	**UIP pattern on CT**	**New GGOs on CT**	**Comorbidities**	Two-peaked deterioration^*^	**Treatment**	**Outcome^†^**
Lee et al. (11)	76	Male	N/A	IPF	Pirfenidone	Yes	Yes	None	No	mPSL, antibiotics, lopinavir/ritonavir, HCQ	Deceased
Rajasurya et al. (16)	79	Female	N/A	IPF	Nintedanib	N/A	N/A	Hypertension	No	Antibiotics, HCQ, tocilizumab, hydrocortisone	Deceased
Akram (17)	60	Male	Ex	IPF	None	Yes	Yes	None	No	HCQ, antibiotics, hydrocortisone, heparin	Deceased
Uzel et al. (18)	64	Male	Ex	IPF	Nintedanib	Yes	Yes	None	No	HCQ, antibiotics, enoxaparin	Survived
Caradec et al. (19)	69	Male	Ex	IPF	Pirfenidone	Yes	Yes	Hypertension, atrial fibrillation, chronic urticaria	No	Antibiotics, HCQ, ruxolitinib	Survived
Omote et al. (12)	87	Female	N/A	IPF	None	Yes	Yes	None	No	mPSL	Survived
Kondoh et al. (8)	73	Male	Ex	NSIP	N/A	N/A	N/A	Hypertension, Parkinson's diease	N/A	N/A	Survived
	85	Male	Never	IPF	N/A	N/A	N/A	Hypertension, hyperlipidemia	N/A	N/A	Deceased
	83	Male	Current	CPFE	N/A	N/A	N/A	Hypertension, IHD	N/A	N/A	Deceased
	64	Female	N/A	CPFE	N/A	N/A	N/A	CI, multiple sclerosis	N/A	N/A	Survived
	69	Female	N/A	IPF	N/A	N/A	N/A	Depression	N/A	N/A	Deceased
	78	Male	Never	IPF	N/A	N/A	N/A	None	N/A	N/A	Deceased
	68	Male	Current	IPF	N/A	N/A	N/A	DM	N/A	N/A	Deceased
	82	Male	Never	NSIP	N/A	N/A	N/A	Dementia, CI, after prostate cancer treatment	N/A	N/A	Deceased
	80	Male	Ex	NSIP	N/A	N/A	N/A	Hypertension, DM, pleural mesothelioma	N/A	N/A	Survived
	72	Male	Current	CPFE	N/A	N/A	N/A	DM	N/A	N/A	Deceased
	73	Male	Ex	RA-ILD	N/A	N/A	N/A	RA	N/A	N/A	Deceased
	73	Male	Ex	IPF	N/A	N/A	N/A	Hypertension	N/A	N/A	Deceased
Fonseca et al. (20)	60	Female	Ex	RA-ILD	None	Yes	Yes	DM, OSA, HF	Yes (N/A)	Azathioprine, HCQ, mPSL	Survived
Earl et al. (21)	79	Female	Never	IPF	None	Yes	No	Hypertension, hyper- cholesterolaemia	Yes (1 month)	Antibiotics, DEX, mPSL, prednisolone	Survived
Present case	60	Male	Ex	IPF	Nintedanib	Yes	Yes	Pulmonary tuberculosis, CI, DM	Yes (1 month)	Antibiotics, DEX, favipiravir, mPSL, prednisolone	Survived

* *The definition of two-peaked deterioration is a case of acute respiratory failure suggestive of worsening or acute exacerbation of ILD after recovery from COVID-19. Periods in the parentheses represent intervals between the diagnosis of preceding COVID-19 and following acute respiratory failure suggestive of worsening or acute exacerbation of ILD*.

† *Kondoh et al. reported 90-day survival of each case*.

*ILD, interstitial lung disease; UIP, usual interstitial pneumonia; GGO, ground-glass opacity; IPF, idiopathic pulmonary fibrosis; NSIP, non-specific interstitial pneumonia; CPFE, combined pulmonary fibrosis with emphysema; RA-ILD, ILD associated with rheumatoid arthritis; DM, diabetes mellitus; IHD, ischemic heart disease; CI, cerebral infarction; RA, rheumatoid arthritis; OSA, obstructive sleep apnea; HF, heart failure; mPSL, methylprednisolone; HCQ, hydroxychloroquine; N/A, not available*.

The patient successfully recovered from the initial phase of acute respiratory failure due to treatment with the anti-viral agent favipiravir (22) and dexamethasone (23), which was the state-of-art treatment regimen at the time in Japan. Moreover, administration of the antifibrotic nintedanib was continued, though the secondary development of AE-IPF was not prevented. As none of vaccines were available at the time in Japan, the patient was not vaccinated against SARS-CoV-2. It is possible that optimal vaccination may reduce the occurrence of AE triggered by COVID-19 in patients with ILD. Future studies are warranted to develop the strategy for preventing and treating AE in IPF patients who developed COVID-19. The optimal duration of anti-viral/anti-inflammatory therapy and/or close monitoring for recovering COVID-19 cases with pre-existing ILD remains unknown. According to the previous reports, the intervals between known triggers and development of AE-IPF ranged between 3–41 days after bronchoalveolar lavage procedures (24) and 0–29 days after lung surgery (25). Considering the experience of the present case, we would emphasize the importance of careful monitoring of patients with IPF after recovery from COVID-19. When patients with pre-existing ILD experience worsening of respiratory failure in the recovery phase of COVID-19, we should consider the possibility of AE-ILD and initiate additional interventions such as corticosteroid pulse therapy.

## Data Availability Statement

The raw data supporting the conclusions of this article will be made available by the authors, without undue reservation.

## Ethics Statement

Written informed consent was waived by the Institutional Review Board, therefore not obtained from the individual. Oral consent was given for the publication of any identifiable information.

## Author Contributions

YG and KS analyzed and interpreted the patient data and drafting the figures. YG, KS, JF, AS, NO, and YS edited the manuscript. All authors have read and approved the final manuscript.

## Funding

This report was partly supported by funding from the Nitto Foundation.

## Conflict of Interest

The authors declare that the research was conducted in the absence of any commercial or financial relationships that could be construed as a potential conflict of interest.

## Publisher's Note

All claims expressed in this article are solely those of the authors and do not necessarily represent those of their affiliated organizations, or those of the publisher, the editors and the reviewers. Any product that may be evaluated in this article, or claim that may be made by its manufacturer, is not guaranteed or endorsed by the publisher.
